# The impact of COVID‐19 on mortality, length of stay, and cost of care among patients with gastrointestinal malignancies: A propensity score‐matched analysis

**DOI:** 10.1002/cam4.6355

**Published:** 2023-07-31

**Authors:** Mark B. Ulanja, Bryce D. Beutler, Kwabena Oppong Asafo‐Agyei, Samuel B. Governor, Samuel Edusa, Daniel Antwi‐Amoabeng, Reginald N. Ulanja, Grace B. Nteim, Millicent Amankwah, Vijay Neelam, Ganiyu A. Rahman, Francis T. Djankpa, Tarig Mabrouk, Olatunji B. Alese

**Affiliations:** ^1^ CHRISTUS Ochsner St. Patrick Hospital Lake Charles Louisiana USA; ^2^ Department of Radiology, Keck School of Medicine University of Southern California Los Angeles California USA; ^3^ CHRISTUS Highland Medical Center Shreveport Louisiana USA; ^4^ School of Medicine Washington University in St Louis St Louis Missouri USA; ^5^ Samalla Clinic Ltd. Vinings Ghana; ^6^ Department of Physiology, School of Medical Sciences University of Cape Coast Cape Coast Ghana; ^7^ Department of Hematology Oncology Feist‐Weiller Cancer Center, Louisiana State University Health Shreveport Shreveport Louisiana USA; ^8^ Department of Surgery, School of Medical Sciences University of Cape Coast Cape Coast Ghana; ^9^ Department of Hematology and Oncology Winship Cancer Institute, Emory University Atlanta Georgia USA

**Keywords:** COVID‐19, gastrointestinal cancer, healthcare cost disparity, impact of COVID‐19 on gastrointestinal malignancies, mortality, propensity score‐matched analysis, SARS‐CoV‐2

## Abstract

**Background:**

Severe acute respiratory syndrome coronavirus 2 (SARS‐CoV‐2) and the coronavirus 19 (COVID‐19) pandemic have had a lasting impact on the care of cancer patients. The impact on patients with gastrointestinal (GI) malignancies remains incompletely understood. We aimed to assess the impact of COVID‐19 on mortality, length of stay (LOS), and cost of care among patients with GI malignancies, and identify differences in outcomes based on primary tumor site.

**Methods:**

We analyzed discharge encounters collected from the National Inpatient Sample (NIS) between March 2020 and December 2020 using propensity score matching (PSM) and COVID‐19 as the treatment effect.

**Results:**

Of the 87,684 patient discharges with GI malignancies, 1892 were positive for COVID‐19 (C+) and eligible for matching in the PSM model. Following PSM analysis, C+ with GI tumors demonstrated increased incidence of mortality compared to their COVID‐19‐negative (C‐) counterparts (21.3% vs. 11.9%, *p* < 0.001). C+ patients with colorectal cancer (CRC) had significantly higher mortality compared to those who were C‐ (40% vs. 24%; *p* = 0.035). In addition, C+ patients with GI tumors had a longer mean LOS (9.4 days vs. 6.9 days; *p* < 0.001) and increased cost of care ($26,048.29 vs. $21,625.2; *p* = 0.001) compared to C‐ patients. C+ patients also had higher odds of mortality secondary to myocardial infarction relative to C‐ patients (OR = 3.54, *p* = 0.001).

**Conclusions:**

C+ patients with GI tumors face approximately double the odds of mortality, increased LOS, and increased cost of care compared to their C‐ counterparts. Outcome disparities were most pronounced among patients with CRC.

## 1 INTRODUCTION

Severe acute respiratory syndrome coronavirus 2 (SARS‐CoV‐2) and the coronavirus disease 2019 (COVID‐19) pandemic have had a lasting impact on the care of cancer patients. In the United States alone, there were over 3.3 million deaths from cancer, COVID‐19, and other causes in 2020, which was almost sixfold the death toll of the previous year; COVID‐19 was responsible for two thirds of the increased deaths.^1^ Approximately 2.0% of cancer deaths in the United States in 2020 were related to COVID‐19. In 2021, that number increased to 2.4%.^2^ Multiple studies have shown that individuals with cancer are at high risk of serious complications from COVID‐19, including thrombosis, debility, and long‐term respiratory complications.^3^, ^4^ Male sex, obesity, increased age, and hematologic and solid malignancies have been identified as poor prognostic factors.^5^, ^6^, ^7^

It has been previously reported that SARS‐CoV‐2 enters the respiratory and gastrointestinal (GI) tracts via the angiotensin‐converting enzyme 2 (ACE2) receptor, causing direct organ damage.^8^ Indeed, SARS‐CoV‐2 has been shown to be present in feces.^9^, ^10^, ^11^ Wang et al. evaluated the expression of infection core genes ACE2 and transmembrane protease serine 2 (TMPRSS2) and noted that the mRNA expression of ACE2 and TMPRSS2 was higher in colorectal cancers (CRC) as compared to other types of cancers.^12^ TMPRSS2 and ACE2 promote entry of the virus into host cells and may lead to direct cellular damage. A subsequent meta‐analysis showed that the case fatality rate among COVID‐19‐positive patients with GI tumors is up to 24%, which is significantly higher than that of the general population.^13^ However, the study was limited by many factors, including publication bias, relatively small sample size, and incomplete data regarding clinical characteristics, prognosis, and cause of death.

The impact of COVID‐19 on patients with GI malignancies remains an important research question, as cancers of the GI tract are common and implicated in greater cancer‐related mortality than all other malignancies combined.^14^, ^15^ Furthermore, despite declining disease‐specific death rate for cancers from 2019 to 2020, deaths from pancreatic cancer (the third leading cause of cancer death in men and women combined) has continued to increase.^1^ We aimed to assess the impact of COVID‐19 infection on mortality, length of stay (LOS), and cost of care among patients with GI malignancies and identify differences in outcomes based on primary tumor site.

## 2 MATERIALS AND METHODS

### 2.1 Study design

The National Inpatient Sample (NIS) is an administrative database of the Healthcare Cost and Utilization Project (HCUP) of the Agency of Healthcare Research and Quality (AHRQ). Patients with GI malignancy who were diagnosed with COVID‐19 from March 1, 2020, to December 31, 2020, were included in the analysis. This is the most current data that are publicly available. The NIS comprises complex survey design with stratified sample of 20% of all discharge encounters involving community hospitals in the United States.

### 2.2 Study population

The discharge encounters of patients aged 18 years or older with a diagnosis of GI malignancy of the following sites were used: esophagus, stomach, small bowel, colorectal, anal, hepatobiliary, and pancreas. Gastrointestinal stromal tumors (GISTs) were also included. The hepatobiliary tumors included hepatocellular carcinoma and cholangiocarcinoma. All discharge encounters were identified using International Classification of Diseases, 10th Edition, Clinical Modification (ICD‐10‐CM). Patients were identified using following site codes; esophagus; C153–C155, C158, C159; stomach; C160–C166, C168–C169, small bowel; C170–C173, C178–C179, colorectal; C180–C189, C19–C20, anal; C210–C212, C218, hepatocellular carcinoma; C220, C222–C224, C227–C229, cholangiocarcinoma; C221, C23, C240–C241, C248–C249, pancreas; C250–C254, C257–C261, GIST; C49A0–C49A5, C49A9. Discharge encounters with secondary malignant neoplasm of GI tract were excluded.

### 2.3 Study variables and outcome measures

The primary outcomes were mortality, LOS, and cost of care. Cost of care was determined by multiplying the “Total Charge” variable in the NIS database to cost‐to‐charge ratio (CCR) to get the actual cost of care paid by the patient or insurance for the hospitalization. Other outcomes of interest included the impact of COVID‐19 on the incidence and co‐mortality of pulmonary embolism, myocardial infarction, and liver failure in patients with GI malignancies. Mortality was calculated as a categorical variable, while LOS and cost of care were on a continuous scale. Patients' sociodemographic characteristics including age, sex, race, and payer type were obtained from Clinical Classifications Software Refined (CCSR) for ICD‐10‐CM Diagnoses (www.hcupus.ahrq.gov/toolssoftware/ccsr/ccs_refined.jsp). Missing observations on key variables including race and sex, age, COVID‐19, and month of diagnosis were excluded. Hospital location and median household income for zip code were also extracted. The Charlson comorbidity index was used to calculate comorbidity index for the analysis. Surgical procedures and chemotherapy treatment were extracted using CCS for International Classification of Diseases, 10th Edition, Procedure Coding System (ICD‐10‐PCS) (www.hcup‐us.ahrq.gov/toolssoftware/ccs10/ccs10.jsp).

### 2.4 Statistical analysis

We used descriptive statistics to summarize demographic and clinical characteristics. Chi‐square test was used to compare categorical variables, while *t*‐test was used for continuous variables. The main outcome analysis was done using logistic regression for mortality, and linear regressions for LOS and cost of care after matching. Missing values for any variable were excluded in the regression models. *p*‐values were two‐sided, and *p* < 0.05 was deemed statistically significant.

### 2.5 Propensity score matching

Propensity score is the probability of group assignment conditional on observed baseline covariates.^16^ To achieve comparable risk groups, propensity score‐matched (PSM) analysis was performed between COVID‐19 positive and COVID‐19 negative cohorts of GI malignancy discharges. Matched pairs had similar values of propensity scores. Matching was done using nearest neighbor matching with 1:1 matching, where the COVID‐19 positive discharge was matched to the COVID‐19 negative counterparts. The propensity score model was calculated using treatment effect (“teffect”) in STATA with the following variables; age, race, month of diagnosis, the Charlson comorbidity index, cancer stage (metastatic vs. nonmetastatic), obesity, history of substance abuse, hypertension, sepsis, mechanical ventilation, community‐acquired pneumonia, surgery and surgical complications, inflammatory bowel disease, history of smoking, insurance status, median household income of residents in the affected ZIP Code, hospital location and academic/teaching status, hospital region, admission status (elective vs. nonelective) and use of palliative care. The discharge encounters were exact matched on gender. COVID‐19 status was considered the treatment in the PSM model. In the PSM model using “teffects,” the average treatment effect of LOS was assessed with other matched variables and thus there was no need to add LOS as a variable in determining the cost of care. All variables deemed necessary were matched; in determining mortality, LOS, and cost of care outcomes, there was no need to further adjust. To assess for adequacy of our matching variables, standardized differences were calculated to compare patient characteristics before and after matching to assess for imbalances. Variables were considered imbalanced if the absolute standardized difference value was greater than 0.10 (small effect size).^16^ All analyses were performed using STATA, version 16.1(Stata Corp).

## 3 RESULTS

**FIGURE 1:**
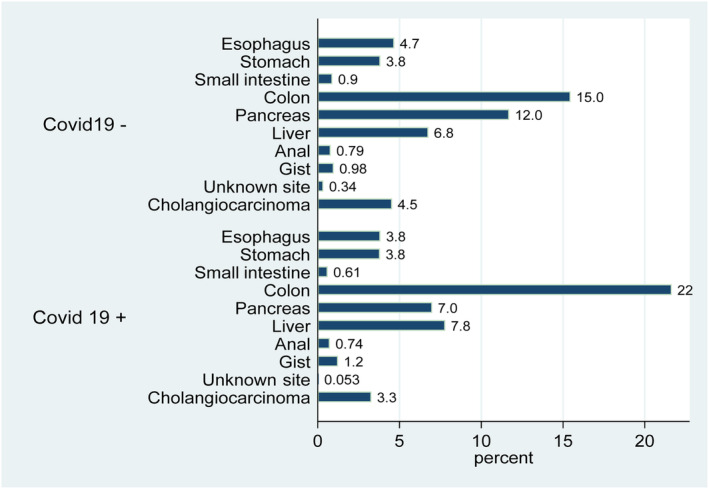
Distribution of primary tumor sites in patients with and without COVID‐19.

Of the 87,684 patient discharges with GI malignancies, 1892 were positive for COVID‐19 (C+) and eligible for matching in the PSM model. Proportion for C+: male (61.2%), female (38.8%). Racial distribution was non‐Hispanic Whites (55.8%), Black (18.0%), Hispanic (18.6%), and Others (Asian, Pacific Islander/Alaskan Natives) (7.6%). The median age was 69 years (IQR: 61–77). The distribution of primary tumor site varied between C+ and C− patients (Figure 1). Monthly variation reflected the wave and plateau dynamics of the spread of COVID‐19. The characteristics of before and after matching cohorts are shown in Table 1.

**TABLE 1:** Characteristics of patients with GI malignancies: before and after propensity score matching (PSM).

Characteristics	Unmatched group				Matched group			
	COVID‐19(−), *n* (%), *N* = 85,792	COVID‐19, *n* (%) (+), *N* = 1892	*p*‐value	Standardized difference	COVID‐19(−) *n* (%), *N* = 1892	COVID‐19 (+) *n* (%), *N* = 1892	*p*‐value	Standardized difference
Age								
18–30	581 (0.7)	^a^	<0.001	0.13	^a^	^a^	0.180	0.01
31–40	2094 (2.4)	38 (2.0)			29 (1.5)	38 (2.0)		
41–50	6485 (7.6)	117 (2.2)			105 (5.6)	117 (6.2)		
51–60	16,283 (19.0)	308 (16.3)			309 (16.3)	308 (16.3)		
61–70	25,983 (30.3)	539 (28.5)			575 (30.4)	539 (28.5)		
71–80	21,846 (25.5)	546 (28.9)			591 (31.2)	546 (28.9)		
81–90	12,520 (14.6)	334 (17.7)			275 (14.5)	334 (17.7)		
Gender								
Female	36,484 (42.5)	734 (38.8)	0.001		734 (38.8)	734 (38.8)	1.00	
Male	49,308 (57.5)	1158 (61.2)			1158 (61.2)	1158 (61.2)		
Race								
Non‐Hispanic Whites (NHW)	58,590 (68.3)	1056 (55.8)	<0.001	0.21	1148 (60.7)	1056 (55.8)	0.009	0.10
Black	11,599 (13.5)	340 (18.0)			338 (17.9)	340 (18.0)		
Hispanic	8962 (10.5)	353 (18.7)			277 (14.6)	353 (18.6)		
APINA/Others	6641 (7.7)	143 (7.6)			129 (6.8)	143 (7.6)		
Month hospitalized (2020)								
March	8575 (10.0)	54 (2.9)	<0.001	0.35	103 (5.4)	54 (2.9)	<0.001	0.10
April	6996 (8.2)	193 (10.2)			102 (5.4)	193 (10.2)		
May	7948 (9.3)	139 (7.4)			129 (6.8)	139 (7.3)		
June	8668 (10.1)	120 (6.3)			164 (8.7)	120 (6.3)		
July	8967 (10.5)	190 (10.0)			194 (10.3)	190 (10.0)		
August	9041 (10.5)	162 (8.6)			213 (11.3)	162 (8.6)		
September	9174 (10.7)	119 (6.3)			219 (11.6)	119 (6.3)		
October	9233 (10.8)	185 (9.8)			269 (14.2)	185 (9.8)		
November	8457 (9.9)	341 (18.0)			268 (14.2)	341 (18.0)		
December	8733 (10.2)	389 (20.6)			231 (12.2)	389 (20.6)		
Charlson comorbidity index (≥2)	85,792	1892	‐	0.07	1892	1892	‐	0.02
Cancer stage								
Nonmetastatic	50,317 (58.7)	1176 (62.2)	0.003	−0.07	1165 (61.6)	1176 (62.2)	0.741	−0.02
Metastatic	35,475 (41.4)	716 (37.8)			727 (38.4)	716 (37.8)		
Obesity								
No	74,438 (86.8)	1647 (87.0)	0.728	−0.01	1659 (87.7)	1647 (87.0)	0.595	0.02
Yes	11,354 (13.2)	245 (13.0)			233 (12.3)	245 (13.0)		
History of substance abuse								
No	83,845 (97.7)	1849 (97.7)	0.993	0.00	1849 (97.7)	1849 (97.7)	1.000	0.00
Yes	1947 (2.3)	43 (2.3)			43 (2.3)	43 (2.3)		
Hypertension								
No	34,510 (40.2)	662 (35.0)	<0.001	0.11	649 (34.3)	662 (35.0)	0.677	−0.01
Yes	51,282 (59.8)	1230 (65.0)			1243 (65.7)	1230 (65.0)		
Sepsis								
No	72,859 (84.9)	1320 (69.8)	<0.001	0.36	1321 (69.8)	1320 (69.8)	0.976	0.00
Yes	12,933 (15.1)	572 (30.2)			571 (30.2)	572 (30.2)		
Use of mechanical ventilation								
No	83,707 (97.6)	1729 (91.4)	<0.001	0.27	1729 (91.4)	1729 (91.4)	1.000	0.00
Yes	2085 (2.4)	163 (8.6)			163 (8.6)	163 (8.6)		
Community‐acquired pneumonia								
No	80,392 (93.7)	1749 (92.4)	0.034	0.05	1749 (92.4)	1749 (92.4)	1.000	0.00
Yes	5400 (6.3)	143 (7.6)			143 (7.6)	143 (7.6)		
Surgery								
No	71,130 (82.9)	1823 (96.4)	<0.001	−0.45	1839 (97.2)	1823 (96.4)	0.341	0.04
Open surgery	8583 (10.0)	54 (2.9)			40 (2.1)	54 (2.9)		
Laparoscopy	5947 (6.9)	15 (0.8)			13 (0.7)	15 (0.8)		
Both	132 (0.2)	0 (0.0)			‐	‐		
Surgical complications								
No	82,858 (96.6)	1868 (98.7)	<0.001	−0.14	1868 (98.7)	1868 (98.7)	1.000	0.00
Yes	2934 (3.4)	24 (1.3)			24 (1.3)	24 (1.3)		
Inflammatory bowel disease								
No	84,748 (98.8)	1868 (98.7)	0.845	0.01	1868 (98.7)	1868 (98.7)	1.000	0.00
Yes	1044 (1.2)	24 (1.3)			24 (1.3)	24 (1.3)		
Smoking history								
No	63,857 (74.4)	1418 (75.0)	0.621	−0.01	1443 (76.3)	1418 (75.0)	0.381	0.03
Yes	21,935 (25.6)	474 (25.0)			449 (23.7)	474 (25.0)		
Insurance								
Medicare	48,318 (56.3)	1132 (59.8)	<0.001	−0.06	1182 (62.5)	1132 (59.8)	0.188	0.05
Medicaid	9862 (11.5)	252 (13.3)			213 (11.3)	252 (13.3)		
Private	22,900 (26.7)	387 (20.5)			398 (21.0)	387 (20.5)		
Self‐pay	2096 (2.4)	38 (2.0)			39 (2.1)	38 (2.0)		
No charge/Other	2517 (2.9)	80 (4.2)			59 (3.1)	80 (4.2)		
Unknown	99 (0.1)	^a^			^a^	^a^		
Median household income of residents in patient's ZIP Code								
0–25th	22,666 (26.4)	597 (31.6)	<0.001	−0.12	579 (30.6)	597 (31.6)	0.068	0.02
26–50th	22,788 (26.6)	501 (26.5)			528 (27.9)	501 (26.5)		
51–75th	20,061 (23.4)	416 (22.0)			437 (23.1)	416 (22.0)		
76–100th	18,821 (21.9)	343 (18.1)			334 (17.7)	343 (18.1)		
Unknown	1456 (1.7)	35 (1.9)			14 (0.7)	35 (1.9)		
Hospital location (including academic/teaching status)								
Rural	5338 (6.2)	144 (7.6)	0.007	−0.08	129 (6.8)	144 (7.6)	0.501	−0.04
Urban nonteaching	12,686 (14.8)	311 (16.4)			294 (15.5)	311 (16.4)		
Urban teaching	67,768 (79.0)	1437 (76.0)			1469 (77.6)	1437 (76.0)		
Hospital region								
Northeast	17,254 (20.1)	412 (21.8)	0.033	−0.07	353 (18.7)	412 (21.8)	0.092	−0.04
Midwest	18,059 (21.1)	435 (23.0)			458 (24.2)	435 (23.0)		
South	33,258 (38.8)	710 (37.5)			768 (40.6)	710 (37.5)		
West	17,221 (20.1)	335 (17.7)			313 (16.5)	335 (17.7)		
Admission type								
Nonelective	64,503 (75.2)	1786 (94.4)	<0.001	−0.55	1793 (94.8)	1786 (94.4)	0.800	0.02
Elective	21,215 (24.7)	104 (5.5)			98 (5.2)	104 (5.5)		
Unknown	74 (0.1)	^a^			^a^	^a^		
Palliative care								
No	73,137 (85.3)	1479 (78.2)	<0.001	0.18	1485 (78.5)	1479 (78.2)	0.829	0.01
Yes	12,655 (14.8)	413 (21.8)			407 (21.5)	413 (21.8)		

^a^ Cells with observations ≤10 could not be shown per data use agreement, and in accordance with the Healthcare Cost and Utilization Project (HCUP) policy.

After PSM, there was no difference in COVID‐19 positivity between patients with selected conditions such as ischemic bowel, acute pancreatitis, liver failure, myocardial infarction (MI), pulmonary embolism and/or deep vein thrombosis (DVT/PE), history of organ transplant, end stage renal disease (ESRD), and the use of immunotherapy. The proportion of colorectal cancer (CRC) discharge encounters with COVID‐19 infection was significantly higher than their COVID‐19 negative counterparts (43.3% vs 30.9%, *p* < 0.001) (Table 2). After matching, the proportion of discharge encounters with acute cholecystitis was 1.2% (23/1892) in C+ and 0.2% (3/1892) in C‐ discharge encounters.

**TABLE 2:** Clinical characteristics of patients with GI cancers and COVID‐19: after propensity score matching.

Variable	Covid 19 (−)	Covid 19 (+)	P‐ value
GI cancer site			
Esophagus	177 (9.4)	145 (7.7)	<0.001
Stomach	145 (7.7)	144 (7.6)	
Small bowel	34 (1.8)	23 (1.2)	
Colon	585 (30.9)	819 (43.3)	
Pancreas	443 (23.4)	265 (14.0)	
Liver	256 (13.5)	295 (15.6)	
Cholangiocarcinoma	172 (9.1)	124 (6.6)	
Anal	30 (1.6)	28 (1.5)	
GIST	37 (2.0)	47 (2.5)	
GI‐unspecified	13 (0.7)	^a^	
Bowel ischemia			
No	1882 (99.5)	1886 (99.7)	0.360
Yes	^a^	^a^	
Cholecystitis			
No	1869 (98.8)	1889 (99.8)	0.0003
Yes	23 (1.2)	^a^	
Acute pancreatitis			
No	1854 (98.0)	1867 (98.7)	0.133
Yes	38 (2.0)	25 (1.3)	
Liver failure			
No	1773 (93.7)	1776 (93.9)	0.859
Yes	119 (6.3)	116 (6.1)	
Myocardial infarction			
No	1855 (98.0)	1863 (98.5)	0.364
Yes	37 (2.0)	29 (1.5)	
Pulmonary embolism			
No	1794 (94.8)	1800 (95.1)	0.655
Yes	98 (5.2)	92 (4.9)	
Deep vein thrombosis			
No	1778 (94.0)	1798 (95.0)	0.154
Yes	114 (6.0)	94 (5.0)	
History of organ transplant			
No	1875 (99.1)	1878 (99.3)	0.654
Yes	17 (0.9)	14 (0.7)	
End‐stage renal disease			
No	1838 (97.2)	1817 (96.0)	0.085
Yes	54 (2.9)	75 (4.0)	
Use of Immunotherapy			
No	1891 (99.9)	1892 (100.0)	0.360
Yes	^a^	0 (0.0)	
Use of Chemotherapy			
No	1870 (98.8)	1886 (99.7)	0.006
Yes	22 (1.2)	^a^	
Mortality			
Died	225 (11.9)	403 (21.3)	<0.001
Alive	1667 (88.1)	1489 (78.7)	
LOS + SD	6.95 ± 8.00	9.42 ± 13.28	<0.001
Median LOS (IQR)	5 (3–8)	6 (3–12)	<0.001
Cost of care + SD	$21,625.24 ± 33,491.03	$26,048.29 **±** 47152.58	0.001
Disposition			
Home	1297 (68.6)	1038 (54.9)	<0.001
Other facility	347 (18.3)	436 (23.0)	
AMA	21 (1.1)	14 (0.7)	
Other	227 (12.0)	404 (21.4)	

Abbreviations: AMA, against medical advice; IQR, inter quartile range; SD, standard deviation.

^a^ Cells with observations <10 could not be shown per data use agreement, and in accordance with the Healthcare Cost and Utilization Project (HCUP) policy.

Furthermore, following PSM analysis, C+ patients with GI tumors demonstrated increased proportion of mortality compared to their C‐ counterparts (21.3% vs. 11.9%; *p* < 0.001). In a logistic regression analysis, C+ discharge encounters for the entire cohort had two times the odds of death compared with those who are C− (OR = 2.01; 95% CI; 1.68–2.39; *p* < 0.001). C+ patients with CRC had significantly higher mortality compared to those who were C−; 39.95% (161/403) versus 24% (54/225) (*p* = 0.035). Other variables were assessed in COVID‐19 patients. Higher odds of mortality from MI was noted among C+ (OR = 3.54, *p* = 0.001), while mortality due to PE or liver failure was not significantly different between C+ and C‐ groups.

The median LOS for C+ and C− patients was 6 (IQR; 3–12) and 5 days (IQR; 3–8), respectively, while the mean LOS was longer for C+ patients (9.42 days vs. 6.95 days, *p* < 0.001). In a linear regression analysis, patients with COVID‐19 stayed 2.47 days longer (95% CI: 1.78–3.17, *p* < 0.001) than those who were negative for COVID‐19.

On average, the cost of care for C+ discharge encounters was higher than for C− discharges ($26,048.29 vs. $21,625.24, *p* = 0.001). The cost difference was $4423.03 (95% CI; $1816.13–$7029.94: *p* = 0.001; Table 3).

**TABLE 3:** Mortality, length of stay, and cost of care among patients with gastrointestinal malignancies and COVID‐19.^a^

Outcome	95% Confidence interval (95% CI)	*p*‐value
Mortality (OR)	2.01 (1.68–2.39)	<0.001
LOS (days)	2.47 (1.78–3.17)	<0.001
Cost of care (US Dollars)	$4423.03 ($1816.13–$7029.94)	0.001

^a^ Reference = those without COVID‐19.

## 4 DISCUSSION

The coronavirus disease 2019 (COVID‐19) pandemic has had far‐reaching effects on global health and cancer care delivery. Cancer is the second leading cause of death in the United States and is an independent risk factor for both COVID‐19 infection and COVID‐19‐related complications.^6^, ^7^, ^17^, ^18^ Indeed, a single‐center retrospective study reported a 13.5% mortality rate among cancer patients.^19^

### 4.1 Mortality

Our study demonstrated a 21.3% mortality rate among COVID‐19‐positive discharge with GI cancers and two times the odds of death compared to their COVID‐19‐negative counterparts. This finding was consistent with a meta‐analysis that showed an overall 24% case fatality rate in patients with GI tumors who developed COVID‐19.^20^ In addition, CRC has been associated with significantly higher mortality in several studies.^20^

SARS‐CoV‐2 is present in feces^3^ and transmission through the fecal–oral route has been described in addition to the more known respiratory tract.^8^ ACE2 receptors are highly expressed in the GI tract with the greatest concentration in the colon and rectum.^6^ The virus binds to the ACE2 receptors to enter the host cell. After the virus enters the endoplasmic body, there is proteolysis and subsequent activation by cathepsin L (CTSL) and furin. One systematic review of 22 publications reported that GI symptoms occurred in approximately 3%–40.7% of patients infected with COVID‐19. GI manifestations included nausea, vomiting, anorexia, diarrhea, abdominal pain, abdominal distension, and hemorrhage.^21^ The virus is associated with dysregulation of the immune response. There is significant myelosuppression, lymphopenia, low immunoglobulin, and long‐lasting immunosuppression. M1 macrophages are initially activated and are associated with the macrophage‐activating syndrome (MAS), lymphopenia, cytokine storm, and damage to cells.^22^ In addition, further insults from myelosuppression associated with cytotoxic chemotherapy and radiotherapy lead to increased susceptibility to viral infections due to weakening of the immune system.

Patients with COVID‐19 received less chemotherapy compared to their counterparts during hospitalization in our study. During the early days of the pandemic, knowledge regarding the safety of chemotherapy was limited with significant concerns for immunosuppression and increased risk of adverse COVID‐19 infection outcomes. As additional information became available, targeted cancer treatment was thought to be protective.^23^ The administration of systemic cytotoxic, adjuvant, palliative, and maintenance chemotherapy was found not to be associated with increased 30‐day all‐cause mortality during the early days of the pandemic.^24^, ^25^, ^26^ Conversely a recent study from the National COVID Cohort Collaborative (N3C) suggested an increased risk of death with recent cytotoxic therapy, with hormonal therapy being associated with decreased mortality risk.^27^ It has also been reported that reducing the use of high‐dose chemotherapy may be associated with lower risk of developing COVID‐19.^23^ It is possible that holding or withdrawing life‐saving chemotherapy from COVID‐19‐positive patients with cancer prior to wide adoption of vaccines could have led to higher mortality reported in our study. Notably, patients who received palliative care were matched, and there was no difference between COVID‐19‐positive and ‐negative cancer patients in this regard.

Our study demonstrated higher mortality among COVID‐19‐positive patients with GI cancers, which was most pronounced among individuals with CRC. Previously reported disruptions in routine cancer care and preventive cancer screening could have been contributory. Unfortunately, this study is limited in determining if delayed screening could have contributed. Most cancer patients had canceled surgeries and self‐isolation due to perceived vulnerability while consultations were being changed to telemedicine could have resulted in worse outcomes due to significant delays in receiving chemotherapy and preventive measures during the pandemic.^26^ The decreased utilization of cancer services could have also led to delays in diagnosing GI cancers, with increased patient presentation with late stage/advanced cancers. One study using a meta‐analytical model on the effect of stage and mortality on colorectal cancer screening delay demonstrated that delays in colorectal cancer screening beyond 6 months leads to a significantly higher number of advanced stages, and beyond 12 months would increase mortality.^28^ The high differential mortality for cancers of colon, plausibly, may also be due to high expression of ACE2 receptors in this part of GI tract than others. Prospective cohort studies and translational research are needed to confirm this hypothesis.

During pandemics, it is crucial to consider other colon cancer screening techniques to reduce the pressure on endoscopic screening. Computed tomography colonography (CTC), multi‐target stool DNA testing, stool‐based tests such as high‐sensitivity guaiac fecal occult blood test (HSgFOBT) or fecal immunochemical testing (FIT) can be employed safely as recommended by the American Society of Clinical Oncology and American Cancer Society.^29^, ^30^

One major factor with demonstrated reduction in mortality from GI cancers and SARS‐CoV‐2 infection is vaccination. Vaccines have been shown to lower infection rates and effectively reduce the risk of poor outcomes especially for patients with cancer.^31^ This is, despite the observation that patients with cancer sometimes have a poor immunological and serological response to vaccination.^32^, ^33^ There is an increased risk of breakthrough SARS‐CoV‐2 infections in these patients compared to those without cancer.^34^, ^35^ COVID‐19 mortality risk after breakthrough infections have been reported to be 6.7%, compared with 1.3% mortality risk among those without breakthrough infections.^34^ The campaign for booster vaccination has been shown to lower the risk of poor outcomes.^36^ High‐risk populations such as GI cancer patients may benefit from COVID‐19 vaccinations and boosters. However, data are limited and long‐term studies are required to assess the effect of vaccination within this population.

### 4.2 Length of stay and cost of care

Individuals with GI cancer who tested positive for COVID‐19 had a mean hospital stay of 9.42 days, which was significantly longer than those without the infection. This finding aligns with previous studies that reported mean hospital stays of 8.07^37^ and 10 days.^38^ Patients with cancer already face an elevated risk for poor health outcomes such as higher mortality, weakened immune systems, and chemotherapy use.^14^, ^39^, ^40^ COVID‐19 can exacerbate these challenges by inducing cytokine release syndrome and inflammation, prolonging hospitalization.^41^ This extended hospitalization carries additional risks, such as hospital‐acquired infections and DVT/PE which can lead to further morbidity.^42^, ^43^ Prolonged hospitalization also results in delayed recovery and missed opportunities for outpatient‐based chemotherapy, adding to the already high mortality risk which cancer patients face.

The average cost of care for GI cancer patients who tested positive for COVID‐19 at discharge in our study was $26,048.29. In contrast, Abuhelwa et al. reported a higher mean total hospitalization charge of $82,849 for cancer patients, using the same database—NIS.^37^ The Abuhelwa et al. study, however, did not clarify how cost was determined and likely used “Total Charge” in NIS, which does not reflect true cost paid by patient or their payer. Additionally, they included most malignancies in their analysis, distorting the data since other malignancies may carry higher cost burden than GI cancers. Notwithstanding, increased cost of care can negatively impact outcomes for cancer patients. Extended hospital stays lead to increased health‐care expenses, hindering patients and payers' abilities to afford subsequent treatments and related financial toxicity.^44^ Financial burden harms patient well‐being, causing anxiety, depression, lower health‐related quality of life with limited access to necessary medical care.^45^

Limitations of our study include its cross‐sectional nature. Data from community hospitals in USA may not be generalizable to the global population. Also, PSM creates selection bias. Some patients' encounters may represent recurrent encounters and not necessarily a unique patient. Additionally, most of these data predate introduction of COVID‐19 vaccination which would likely have effects on outcomes, especially mortality and LOS. We will be doing a follow‐up study to further asses these outcomes during vaccination era. Despite these limitations, using PSM as a causal inference model provides important insight into the effects of COVID‐19 on GI cancers. Also, the sample size makes the study one of the largest, looking at COVID‐19 and specifically GI cancer mortality, LOS and cost of care.

## 5 CONCLUSION

This PSM analysis suggests that prior to the introduction of vaccines, COVID‐positive patients with GI tumors faced approximately double the odds of mortality, an increased LOS of 2.5 days, and increased cost of care of about $4400 compared to their COVID‐negative counterparts. The disparity of outcomes was most pronounced among individuals with CRC. It is possible that the direct effects of the SARS‐CoV‐2 virus on the GI tract may increase disease severity and mortality. This bolsters the campaign for universal COVID‐19 vaccination, especially among individuals with GI malignancies. The direct and indirect mechanisms underlying increased mortality among COVID‐19‐positive patients with GI malignancies warrant further investigation.

## AUTHOR CONTRIBUTIONS

**Mark Ulanja:** Conceptualization (lead); data curation (equal); formal analysis (equal). **Bryce D. Beutler:** Writing – original draft (lead); writing – review and editing (supporting). **Kwabena Oppong Asafo‐Agyei:** Data curation (equal); formal analysis (equal); investigation (equal). **Samuel Governor:** Data curation (equal); formal analysis (equal); investigation (equal). **Samuel Edusa:** Formal analysis (supporting); investigation (supporting). **Daniel Antwi‐Amoabeng:** Data curation (supporting); formal analysis (supporting); investigation (supporting); methodology (supporting). **Reginald Ulanja:** Data curation (supporting); formal analysis (supporting); methodology (supporting). **Grace B. Nteim:** Writing – review and editing (equal). **Millicent Amankwah:** Data curation (supporting); formal analysis (supporting); investigation (supporting). **Vijay Neelam:** Data curation (equal); formal analysis (equal). **Ganiyu A Rahman:** Supervision (equal); validation (equal); visualization (equal); writing – review and editing (supporting). **Francis Djankpa:** Investigation (supporting); methodology (supporting). **Tariq Mabrouk:** Data curation (equal); formal analysis (equal); funding acquisition (equal). **Olatunji B. Alese:** Conceptualization (equal); supervision (equal); writing – review and editing (equal).

## ETHICS APPROVAL STATEMENT

The data used in this study are publicly available through the National Inpatient Sample and therefore Institutional Board Review approval was not required. The study conforms to all recognized ethics standards.

## Data Availability

Data are publicly available through the National Inpatient Sample (NIS), an administrative database of the Healthcare Cost and Utilization Project (HCUP) of the Agency of Healthcare Research and Quality (AHRQ).
